# Systemic endothelial glycocalyx shedding mediates vascular hyperpermeability after traumatic brain injury

**DOI:** 10.3389/fneur.2025.1706102

**Published:** 2026-01-08

**Authors:** Marcela Curci Vieira de Almeida, Maria Clara Zanon Zotin, Carlos Henrique Miranda

**Affiliations:** 1 Division of Emergency Medicine, Department of Internal Medicine, Ribeirão Preto School of Medicine, University of São Paulo, Ribeirão Preto, SP, Brazil; 2 Department of Medical Imaging, Hematology and Oncology, Ribeirão Preto School of Medicine, University of São Paulo (USP), Ribeirão Preto, SP, Brazil

**Keywords:** endothelial glycocalyx, traumatic brain injury, blood–brain barrier, microalbuminuria, cerebral edema

## Abstract

**Background:**

In traumatic brain injury (TBI), the primary insult initiates a secondary cascade that exacerbates neuronal injury. Blood–brain barrier (BBB) dysfunction plays a central role in this process, leading to vascular leakage and vasogenic edema. Recent evidence suggests that the endothelial glycocalyx (eGC) is an essential structural component of the BBB. This study aimed to determine whether systemic eGC shedding after TBI contributes to vascular hyperpermeability and cerebral edema.

**Methods:**

We enrolled patients within 24 h of TBI. Blood and urine samples were collected to measure biomarkers of eGC shedding [syndecan-1 (SDC-1), soluble CD44 (CD44s), hyaluronan (HA), sulfated glycosaminoglycans (GAGs)], of endothelial cell damage [thrombomodulin (sTM)], of inflammation [interleukin-6 (IL-6)], and of vascular permeability [microalbuminuria]. Neuron-specific enolase (NSE) was measured as a surrogate marker of neuronal injury and BBB disruption. eGC thickness was estimated via sublingual microcirculation capillaroscopy using the perfused boundary region (PBR)—an inverse parameter of eGC thickness. Cranial computed tomography (CT) was used to assess signs of cerebral edema. A modified Rankin Scale (mRS) score ≥4 at 3 months was considered a poor neurological outcome.

**Results:**

We enrolled 55 TBI patients, and 20 healthy individuals served as controls. Compared with controls, TBI patients had significantly higher SDC-1, CD44s, GAGs, sTM, IL-6, NSE, and microalbuminuria levels, as well as higher adjusted PBR values. The levels of SDC-1, CD44s, sTM, IL-6, and microalbuminuria showed a statistically significant correlation with NSE levels. Additionally, a significant positive correlation was observed between microalbuminuria levels and adjusted PBR. Microalbuminuria was higher in those with cistern compression on CT and in those with poor neurological outcomes.

**Conclusion:**

Systemic eGC shedding appears to be an early and central pathophysiological event after TBI, contributing to systemic vascular hyperpermeability and thereby to the development of cerebral edema.

## Introduction

Traumatic brain injury (TBI) remains one of the leading causes of death and long-term disability worldwide, particularly among young adults (1). Despite advances in trauma and neurocritical care, outcomes for moderate and severe TBI have shown little improvement over recent decades (2). The secondary phase—a complex cascade of pathophysiological processes that evolve after the initial mechanical insult—plays a pivotal function in neuronal injury. Blood–brain barrier (BBB) dysfunction, microvascular failure, neuroinflammation, and cerebral edema are critical events in this secondary phase (3, 4). Early identification and modulation of these processes remain major challenges in acute neurocritical care and hold the potential to improve outcomes (5).

The endothelial glycocalyx (eGC) is a carbohydrate–protein layer attached to the endothelial cell membrane that covers the luminal surface of all vessels of the body, including the brain vessels (6, 7). Recent evidence has identified the eGC as a critical structural and functional component of the BBB (8, 9). Clinical and experimental studies have demonstrated that early eGC shedding occurs in conditions such as trauma and sepsis, leading to vascular leakage, inflammatory activation, and tissue edema (10, 11). Recent experimental studies have demonstrated a reduction in eGC thickness following repeated exposures to both low- and high-intensity blasts in rats, with a significant decrease in cerebral blood flow observed after high-intensity exposure (12, 13). Clinical investigations have also reported elevated levels of eGC shedding biomarkers—particularly syndecan-1—in patients following TBI (14, 15). However, scientific evidence directly linking systemic eGC shedding to vascular hyperpermeability and subsequent cerebral edema in this context remains limited. Based on this, the present study aimed to investigate the contribution of eGC shedding following TBI in the development of vascular hyperpermeability and cerebral edema.

## Methods

**Figure 1:**
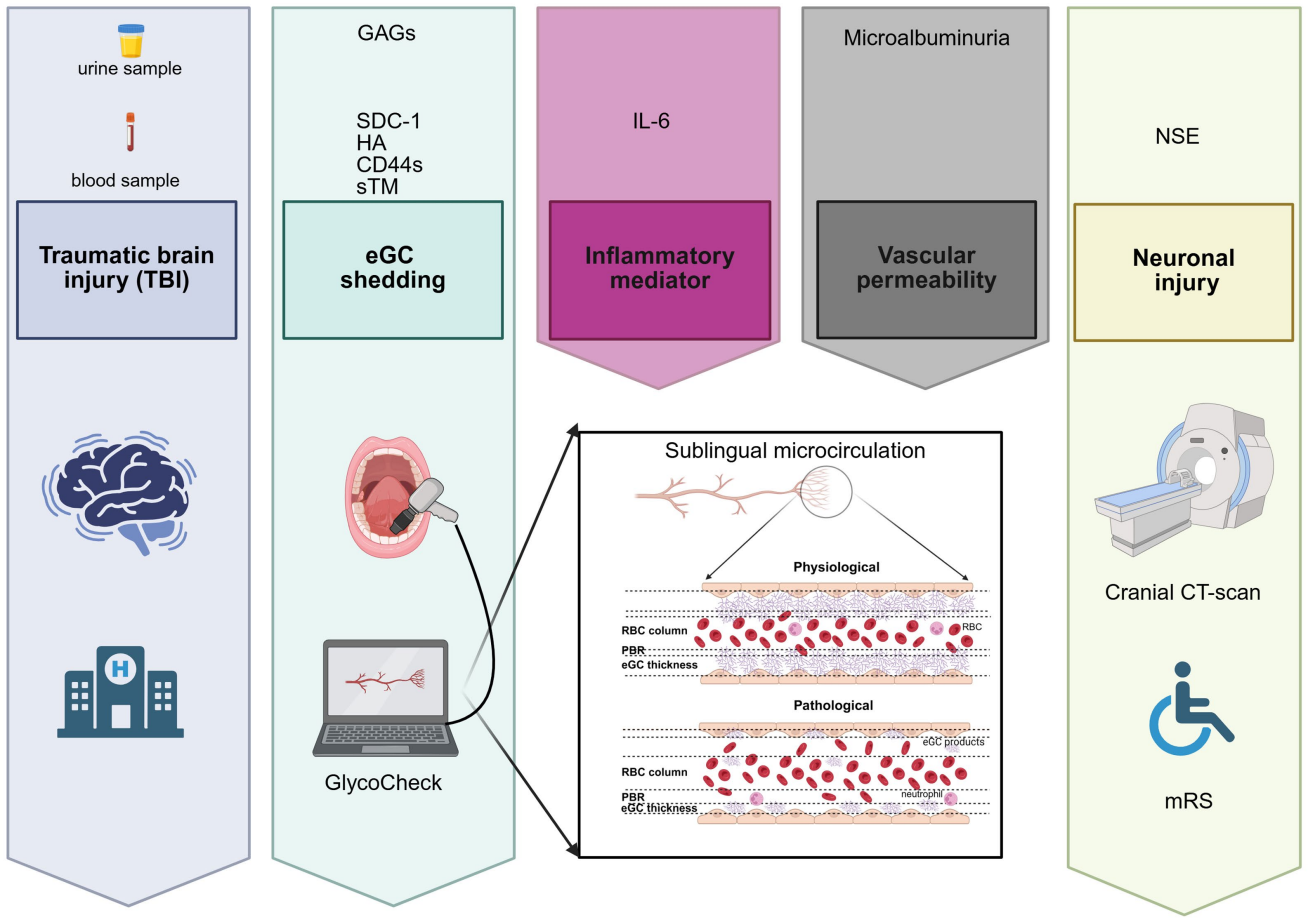
Schematic overview of all laboratory and clinical assessments performed in patients included in the study. eGC, endothelial glycocalyx; SDC-1, syndecan-1; CD44s, antigen soluble CD44; HA, hyaluronan; GAGs, sulfated glycosaminoglycan; sTM, thrombomodulin; IL-6, interleukin-6; NSE, neuron-specific enolase; PBR, perfused boundary region; RBC, red blood cell; mRS, modified Rankin Scale; CT, computed tomography.

This was a single-center, observational, cross-sectional, and prospective study, conducted at a tertiary trauma hospital in Brazil. We evaluated eGC shedding in patients with TBI through the measurement of blood and urinary biomarkers, as well as by sublingual videomicroscopy to estimate eGC thickness using the GlycoCheck software. Figure 1 provides a summary of all clinical and laboratory assessments conducted on the patients included in the study. The research was approved by the Research Ethics Committee from the Hospital das Clínicas of the Ribeirao Preto School of Medicine under the number CAAE 28972620.5.0000.5440. The informed consent form was obtained from all participants or their legal guardians. The research was performed in accordance with the Declaration of Helsinki.

### Patients and healthy individuals

We included patients of all genders, aged over 18 years, hospitalized with TBI within the first 24 h after trauma. The exclusion criteria were mild TBI without indication for cranial computed tomography (CT); prior cardiopulmonary arrest; brain death at admission; patients receiving palliative care; advanced liver failure (Child B or C); stage IV or dialysis-dependent kidney failure; active cancer; major burn with >40% total body surface area; circulatory shock defined by the need for vasoactive drugs to maintain systolic blood pressure > 90 mmHg and arterial lactate ≥2 mg/dL; previous ischemic or hemorrhagic stroke; pre-existing neurological disease; and pregnancy. A control group of 20 healthy individuals was recruited from physicians and nurses from the hospital, aged over 18 years, with no known diseases.

### Clinical laboratory data

General demographic, clinical, and laboratory data were collected from medical records at hospital admission. The following laboratory tests were evaluated: complete blood count, creatinine, urea, sodium, potassium, prothrombin time (PT), activated partial thromboplastin time (aPTT), and fibrinogen. These tests were routinely performed during hospital admission in all TBI patients. TBI severity was classified according to the Glasgow Coma Scale as mild (13–15 points), moderate (9–12 points), and severe (3–8 points). The Corticosteroid Randomization After Significant Head injury trial (CRASH) score, a validated prognostic tool for TBI, was calculated using admission data (16). Overall trauma severity was assessed using the Injury Severity Score (ISS) (17).

### Cranial computed tomography

All cranial CT scans were reviewed by an experienced neuroradiologist. The following parameters were assessed in the initial CT: time from admission to scan, presence and measurement (in millimeters) of midline shift, presence and dimensions of epidural or subdural hematoma, subarachnoid hemorrhage, depressed skull fracture, pneumocephalus, third ventricle, lateral ventricles, or cistern compression, and sulci effacement (18). TBI severity was also classified using the Marshall CT classification (19). We considered midline shift, third ventricle, lateral ventricles, or cistern compression, and sulci effacement as CT signs of cerebral edema.

### Biomarkers

Blood and urine samples were collected in preservative-free tubes at study inclusion and centrifuged at 1,000 × *g* for 10 min in a refrigerated centrifuge. Blood samples showing hemolysis were recollected. Serum and urine were stored at −70 °C. All blood biomarkers were measured by commercial enzyme-linked immunosorbent assay (ELISA) kits from R&D Systems (Minneapolis, USA) according to the manufacturer’s instructions. The biomarkers of eGC shedding measured were the syndecan-1 (SDC-1) (Catalog#: DY2780), CD44 antigen soluble (CD44s) (Catalog#: DY7045-05), and hyaluronan (HA) (Catalog#: DY3614). Thrombomodulin (sTM) (Catalog#: DY3947) was measured as a biomarker of endothelial damage. Interleukin-6 (IL-6) (Catalog#: DY206) was measured as an inflammatory mediator. Neuron-specific enolase (NSE) (Catalog#: DY5169-05) was measured as a surrogate biomarker of neuronal injury and BBB disruption. NSE is normally confined to the cytoplasm of neurons and enters the systemic circulation only when neuronal integrity and endothelial barrier function are compromised (20, 21). Urinary sulfated glycosaminoglycan (GAGs), another biomarker of eGC shedding, was measured using the colorimetric Dimethylmethylene Blue Assay (DMMB). GAG values were normalized to urinary creatinine (Bio-Rad, Irvine, CA, USA). This method estimates total sulfated glycosaminoglycans (GAGs), including heparan sulfate, chondroitin sulfate, and dermatan sulfate (22). SDC-1 serves as the major core proteoglycan, linked to GAGs, while HA, the only non-sulfated glycosaminoglycan, interacts with the glycoprotein CD44s. These are the main structural eGC components (6, 7). In this same urinary sample, microalbuminuria was measured by immunoturbidimetry through the microalbuminuria Turbiquest Plus reagents (Labtest Diagnóstica, Lagoa Santa, Brazil) and normalized to urinary creatinine. Patients with diabetes mellitus (*n* = 2) or hypertension (*n* = 3) were excluded from the microalbuminuria analysis. Microalbuminuria is recognized as a sensitive surrogate marker of vascular permeability because it reflects early, systemic endothelial dysfunction (23). Under physiological conditions, the eGC and the tight junctions of the glomerular filtration barrier restrict albumin passage into the urine. When endothelial integrity is compromised, the vascular barrier becomes more permeable, allowing albumin to leak into the urinary space (24).

### Sublingual microcirculation assessment

Sublingual microcirculation was assessed with a portable videocapillaroscope (Capiscope HCVS, KK Technology, Honiton, UK), consisting of a camera attached to a magnifying lens, inserted non-invasively under the tongue to visualize the local capillary network. Green light at hemoglobin’s absorption wavelength (540 nm) was used to visualize red blood cell (RBC) flow. The images were captured at 5 × optical magnification (325 × total magnification) at 720 × 576 pixels, with 23 frames per second. The images were automatically analyzed using GlycoCheck™ software 2.0 (Microvascular Health Solutions, Alpine, UT, USA), which only records when quality criteria for movement, illumination, and focus are met. Each complete recording consisted of at least ten 2-s videos (~3,000 vascular segments per recording, spaced 10 μm apart). Segments were considered valid if the RBC content was ≥50%, the RBC column was >2 μm, and the segments were not curved or overlapping. Invalid segments were discarded; valid ones were analyzed. Three measurements were taken in different sublingual regions, and the mean was calculated. The software calculates the dynamic lateral displacement of RBCs within the permeable part of the eGC, expressed as the perfused boundary region (PBR), an inversely related parameter to eGC thickness. Because RBC penetration depends on flow velocity, PBR was adjusted for RBC velocity (adjusted PBR). Perfused vascular density (PVD) in mm/mm^2^ was calculated by multiplying the number of valid segments by their length (10 μm) and normalizing by the scanned surface area. The microvascular health score (MVHS) is a composite metric intended to quantify microvascular integrity *in vivo*, especially in critically ill patients, developed by Rovas et al. (25). MVHS integrates two key domains of microvascular health: (a) capillary blood volume/flow and (b) glycocalyx integrity (via the PBR). It ranges from 0 to 10, with lower values indicating greater microvascular impairment.

### Clinical outcomes

Neurological sequelae from TBI were assessed using the modified Rankin Scale (mRS) applied 3 months post-injury. The mRS is a 7-point scale (0–6) that measures global functional disability, with higher scores indicating greater dependence or death. This assessment was conducted via telephone, a validated method for application of this scale in the literature (26). All evaluations were performed by the same observer, who was blinded to patients’ biomarker levels and sublingual microcirculation parameters. Poor neurological outcome was defined as mRS ≥ 4.

### Statistical analysis

A convenience sampling method was used, and a formal sample size calculation was not conducted due to the lack of comprehensive data in the literature—available studies primarily reported on individual biomarkers, particularly SDC-1. The Shapiro–Wilk test assessed the variable distribution. Continuous variables following a normal distribution were presented as mean ± standard deviation (SD) and analyzed using unpaired Student’s *t*-test. Variables with a non-normal distribution were expressed as median and interquartile range (IQR) and analyzed using the Mann–Whitney *U* test. Categorical variables were expressed as frequencies and percentages and compared using the chi-square test. Correlations between quantitative variables were evaluated using Spearman’s non-parametric test. Statistical analyses and graphs were performed using GraphPad Prism 10 (California, USA). A two-tailed *p*-value <0.05 was considered statistically significant.

## Results

**Figure 2:**
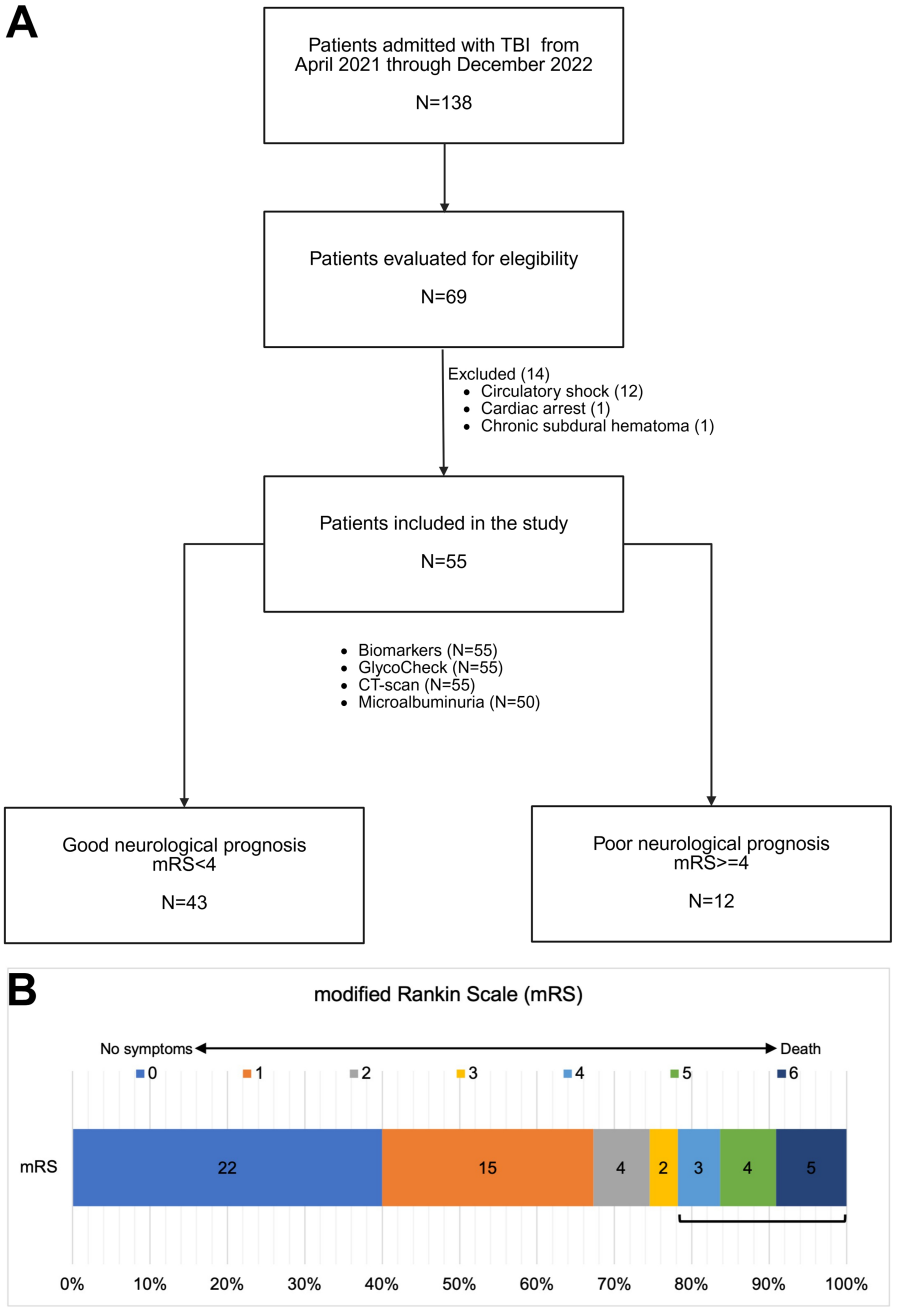
**(A)** Flowchart illustrating patients included in the study. **(B)** Distribution of patients according to the modified Rankin Scale (mRS) scores assessed 3 months post-traumatic brain injury.

**Figure 3:**
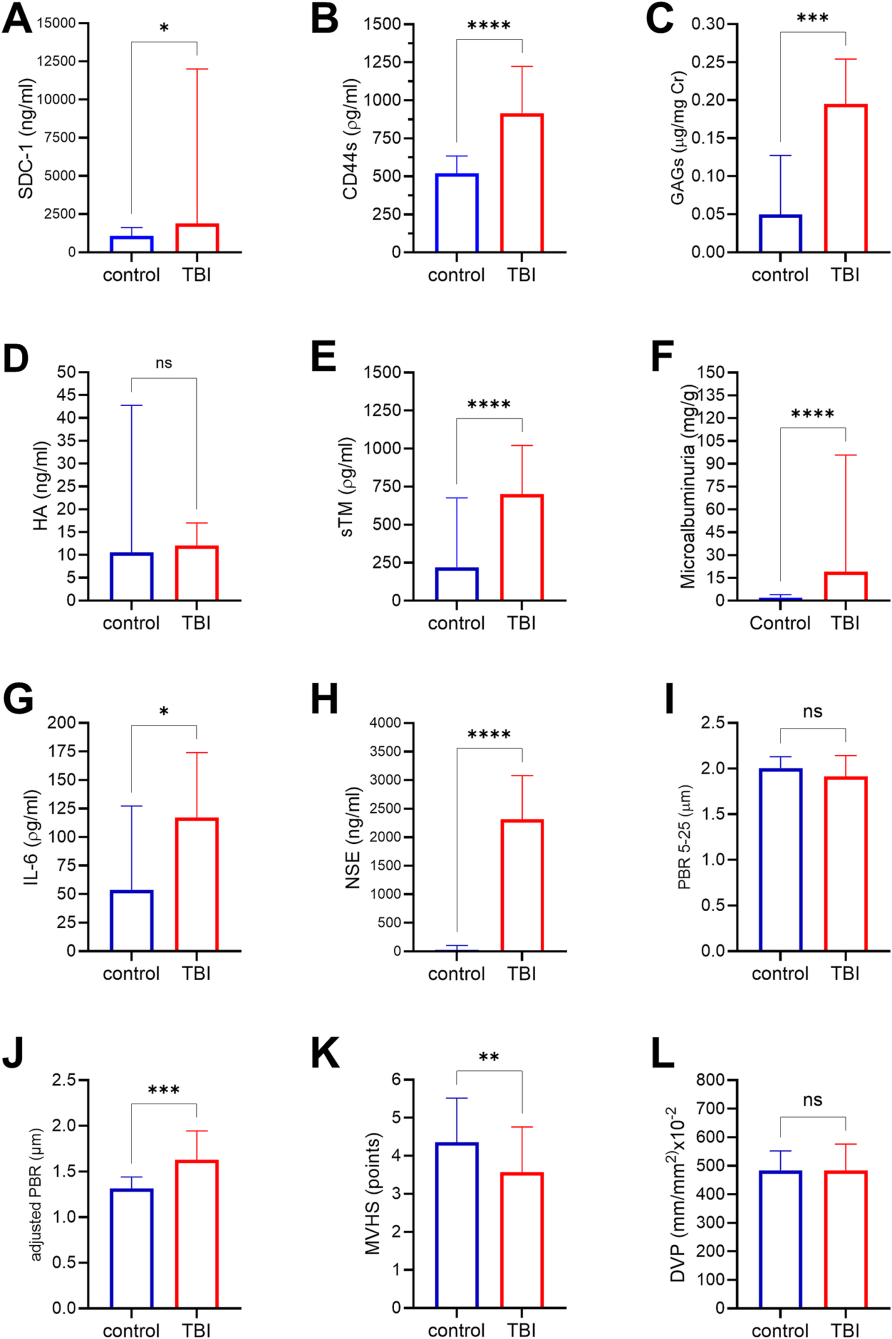
Bar charts comparing levels of biomarkers reflecting endothelial glycocalyx (eGC) shedding **(A–D)**, endothelial cell damage **(E)**, vascular permeability **(F)**, inflammation **(G)**, neuronal injury **(H)**, and sublingual microcirculation parameters between traumatic brain injury (TBI) patients and healthy individuals (control). Data are presented as median and 75th percentile. SDC-1, syndecan-1; CD44s, antigen soluble CD44; GAGs, sulfated glycosaminoglycan; HA, hyaluronan; sTM, thrombomodulin; IL-6, interleukin-6; NSE, neuron-specific enolase; PBR, perfused boundary region; MVHS, microvascular health score; PVD, perfused vascular density; ns: non-significant; *p < 0.05; **p < 0.01; ***p < 0.001; ****p < 0.0001.

**Figure 4:**
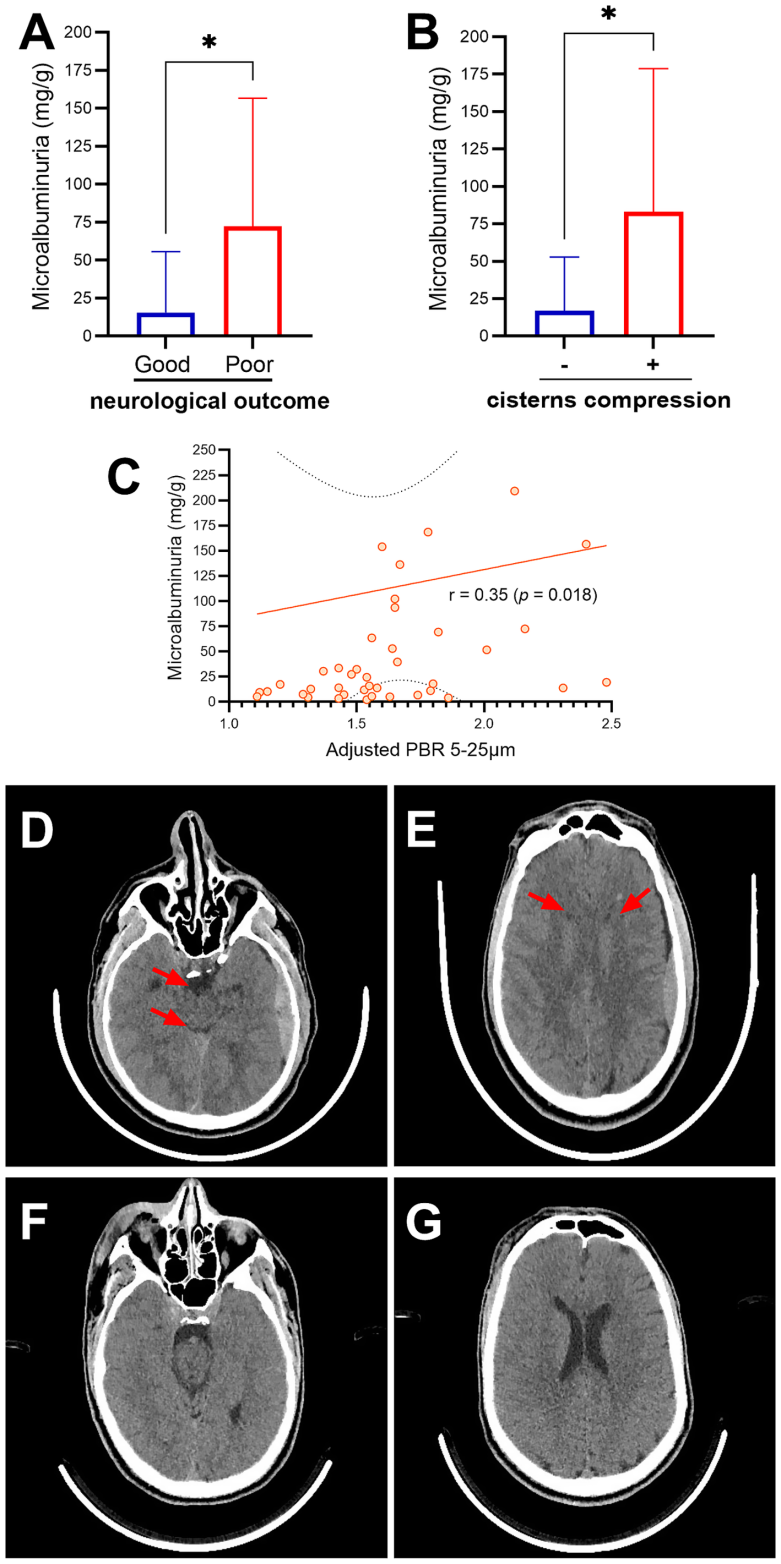
Bar chart comparing microalbuminuria levels between patients with good and poor neurological outcomes **(A)**, and between patients with (+) or without (−) cistern compression on cranial computed tomography (CT) following traumatic brain injury (TBI) **(B)**. Scatter plot showing the correlation between the perfused boundary region (PBR)—an inverse indicator of endothelial glycocalyx (eGC) thickness—and microalbuminuria levels **(C)**. Representative CT images illustrating cistern **(D)** and lateral ventricular **(E)** compression (arrows) in a patient with severe TBI, compared with a patient with mild TBI in whom these spaces are preserved **(F,G)**. *r*, coefficient correlation; *p < 0.05.

**Figure 5:**
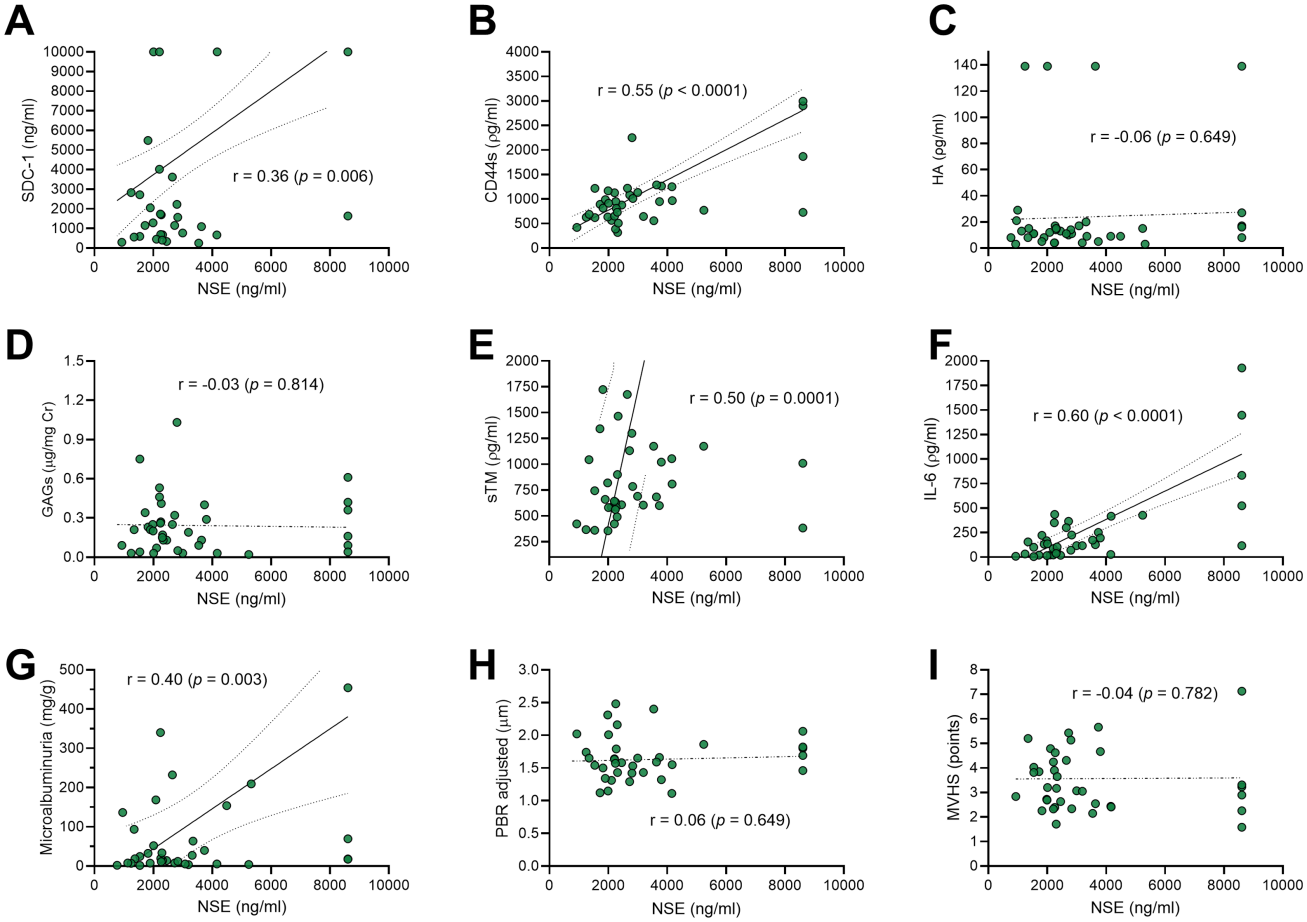
Scatter-plots exhibiting the correlation between the neuron-specific enolase (NSE)—as a surrogate of neuronal injury and blood–brain barrier disruption—and the biomarkers levels of endothelial glycocalyx (eGC) shedding **(A–D)**, endothelial cell damage **(E)**, inflammation **(F)**, vascular permeability **(G)**, and sublingual microcirculation parameters **(H,I)** in traumatic brain injury (TBI). SDC-1, syndecan-1; CD44s, antigen soluble CD44; GAGs, sulfated glycosaminoglycan; HA, hyaluronan; sTM, thrombomodulin; IL-6, interleukin-6; PBR, perfused boundary region; MVHS, microvascular health score; *r*, correlation coefficient.

From 1 April 2021 to 31 December 2022, a total of 138 patients with TBI were admitted to the hospital. Of these, 69 patients were recruited for this study. Totally, 14 patients were subsequently excluded for the following reasons: 12 had circulatory shock, one experienced cardiac arrest, and one had an acute exacerbation of a chronic subdural hematoma. Consequently, 55 patients were included in the final analysis. The flowchart of the patients included in this study is shown in Figure 2A. Figure 2B shows the mRS distribution of these patients 3 months after the trauma; 12 of the 55 (22%) patients had a poor neurological outcome. The baseline demographic, clinical, and laboratory characteristics of the patients included in the study are shown in Table 1. The majority of the patients were male (49/55, 89%), with a mean age of 38 ± 14 years. The severity of TBI was severe in 32 of the 55 (58%) patients, mild in 16 of the 55 (29%) patients, and moderate in 7 of the 55 (13%) patients. The majority of the patients had other associated injuries (39/55, 71%). The mean ISS was 17 ± 9. The median time from trauma to hospital admission was 159 min (IQR 60–254). The CRASH score showed a value of 11 (IQR 3–22), corresponding to a predicted 6-month unfavorable outcome of 30% (IQR 12–59). For comparison, we included 20 healthy individuals with a similar age of 35 ± 14 years to the TBI patients (*p* = 0.420), but with a lower prevalence of male gender (10/20, 50%). The cranial CT parameters analyzed, stratified according to the poor or good neurological prognosis at 3 months, are shown in Table 2. Regarding eGC shedding biomarkers, SDC-1 [1894 ng/mL (IQR 745–12,000) vs. 1,074 ng/mL (IQR 352–1,431); *p* = 0.03], CD44s [916 ρg/mL (IQR 645–1,223) vs. 519 ρg/mL (IQR 317–634); *p* < 0.0001], and GAGs levels [0.20 ρg/mg (IQR 0.09–0.31) vs. 0.05 ρg/mg (IQR 0.02–0.13); *p* = 0.0007] were statistically higher in the TBI group than those in controls. There was no difference between the groups regarding HA levels (Figures 3A–D). The sTM [701 ρg/mL (IQR 580–1,175) vs. 220 ρg/mL (IQR 63–677); *p* < 0.0001] and IL-6 levels [117.0 ρg/mL (IQR 37.0–297.0) vs. 53.5 ρg/mL (IQR 10.5–127.3); *p* = 0.04] were significantly higher in the TBI patients than in the controls (Figures 3E,G). The NSE levels were markedly elevated in TBI patients [2,316 ng/mL (IQR 1897–3,736) vs. 29 ng/mL (IQR 29–104); *p* < 0.0001] compared with controls (Figure 3H). Regarding vascular permeability, microalbuminuria was significantly higher in TBI patients [19.01 mg/g (IQR 8.95–95.78)] than in controls [2.08 mg/g (IQR 1.05–4.06); *p* < 0.0001] (Figure 3F). For sublingual microcirculation parameters, the adjusted PBR, an inverse parameter of eGC thickness, was significantly higher in TBI patients [1.57 μm (IQR 1.41–1.79)] than in controls [1.32 μm (IQR 1.18–1.44); *p* = 0.0002], and the microvascular health score (MVHS) was significantly lower in the TBI group [3.36 points (IQR 2.68–4.68)] than in controls [4.36 points (IQR 3.74–5.51); *p* = 0.005], reinforcing the systemic eGC shedding and microcirculation impairment in TBI patients (Figures 3J,K). There were no statistically significant differences between the groups for unadjusted PBR (*p* = 0.33) and for PVD (*p* = 0.823) (Figures 3I,L). All the aforementioned analyses were repeated with the exclusion of patients with hypertension and diabetes, and the results remained consistent with the original findings. Furthermore, a statistically significant correlation was observed between microalbuminuria levels and adjusted PBR (*r* = 0.35; *p* = 0.018) and MVHS (*r* = −0.31; *p* = 0.040) (Figure 4). There was a statistically significant positive correlation between NSE levels—used as a surrogate marker of neuronal injury and BBB disruption—and several vascular or inflammatory markers, including SDC-1 (*r* = 0.36, *p* = 0.006), CD44s (*r* = 0.55, *p* < 0.0001), sTM (*r* = 0.50, *p* = 0.0001), IL-6 (*r* = 0.60, *p* < 0.0001), and microalbuminuria (*r* = 0.40, *p* = 0.003). In contrast, no significant correlations were observed between NSE levels and HA, GAGs, adjusted PBR, or MVHS (Figure 5). We analyzed the biomarker levels and sublingual microcirculation parameters according to the presence or absence of cranial CT signs of cerebral edema (midline shift, third ventricle, lateral ventricles, and cistern compression or sulci effacement). Despite higher SDC-1, CD44s, and sTM levels in patients with these signals, only the microalbuminuria levels achieve statistical significance, with higher levels in patients with cistern compression [83.05 mg/g (IQR 39.60–168.54) vs. 17.24 mg/g (IQR 7.36–52.32); *p* = 0.017] (Table 3 and Figure 4). Although NSE levels did not reach statistical significance, they were higher in patients exhibiting midline shift, compression of the third and lateral ventricles, cistern compression, or sulcal effacement, reinforcing their utility as a surrogate marker of neuronal injury and BBB disruption (Table 3).

**Table 1:** Baseline demographic, clinical, and laboratory characteristics of patients with traumatic brain injury (TBI) and healthy controls.

Characteristics	TBI	Control	*p*-value
	*N* = 55	*N* = 20	
Demographics			
Age; years, mean ± sd	38 ± 14	35 ± 14	0.420
Gender; male, *n*(%)	49(89)	10(50)	0.0007
Comorbidities; *n*(%)			
Diabetes mellitus	2(04)	–	
Hypertension	3(06)	–	
Mechanism of injury; *n*(%)			
Motorcycle accident	21(38)	–	
Fall greater than 2 m	9(16)	–	
Bicycle accident	8(15)	–	
Physical aggression	7(13)	–	
Automobile accident	3(05)	–	
Fall lesser than 2 m	2(04)	–	
Other	5(09)	–	
Clinical presentation			
Symptoms; *n*(%)	35(64)	–	
Lowering of the level of consciousness; *n*(%)	7(13)	–	
Mental confusion; *n*(%)	7(13)	–	
Transient loss of consciousness; *n*(%)	3(5)	–	
Glasgow Coma Scale; median(IQR)	9(5–14)	–	
Severity; *n*(%)			
Mild	16(29)	–	
Moderate	7(13)	–	
Severe	32(58)	–	
Trauma			
Extracranial trauma; *n*(%)	39(71)	–	
ISS; mean ± sd	17 ± 9	–	
Time since injury; minutes, median(IQR)	159(60–254)		
Vital signals; mean ± sd			
Systolic blood pressure; mmHg	125 ± 23	–	
Diastolic blood pressure; mmHg	79 ± 15	–	
Heart rate; bpm	88 ± 18	–	
Respiratory rate; ipm	20 ± 3	–	
Oxygen arterial saturation; (%)	98 ± 5	–	
Lab tests; mean ±sd			
Hemoglobin; g/dL	13.75 ± 1.66	–	
Hematocrit; (%)	42 ± 5	–	
Leukocytes; /mm^3^	15,000 ± 5,600	–	
Platelets; /mm^3^	245,000 ± 83,000	–	
Creatinine; mg/dL	1.03 ± 0.29	–	
Urea; mg/dL	31.74 ± 11.20	–	
Sodium; mmol/L	138 ± 4.15	–	
Potassium; mmol/L	3.88 ± 0.52	–	
Prothrombin time (PT), INR	1.11 ± 0.24	–	
aPTT, ratio	1.06 ± 0.41	–	
Fibrinogen; mg/dL	258 ± 85	–	
Treatment			
Mechanical ventilation; *n*(%)	34(62)	–	
Sedation; *n*(%)	36(65)	–	
Propofol; *n*(%)	30(55)	–	
Midazolam; *n*(%)	6(11)	–	
Fentanyl; *n*(%)	35(64)	–	
Anticonvulsant; *n*(%)	31(56)	–	
Tranexamic acid; *n*(%)	4(7)	–	
Scores			
CRASH; points, median(IQR)	11(3–22)	–	
CRASH; (%), median(IQR)	30(12–59)	–	
Marshall tomographic classification; *n*(%)			
Category I	28(51)	–	
Category II	20(36)	–	
Category III	6(11)	–	
Category IV	1(2)	–	

sd, standard deviation; IQR, interquartile range; aPTT, activated partial thromboplastin time; ISS, Injury Severity Score; CRASH, Corticosteroid Randomization After Significant Head injury score.

**Table 2:** Cranial computed tomography (CT) findings categorized according to neurological outcomes assessed by the modified Rankin Scale (mRS) 3 months after traumatic brain injury (TBI).

	Total	Good neurological outcomes (mRS < 4)	Poor neurological outcomes (mRS ≥ 4)	*p*-value
	*N* = 55	*N* = 43	*N* = 12	
Midline shift (MLS)				
MLS; *n*(%)	8 (14.5)	5 (11.6)	3 (25.0)	0.245
MLS 0–5 mm; *n*(%)	4 (4.3)	3 (7.0)	1 (8.3)	0.873
MLS > 5 mm; *n*(%)	4 (7.3)	2 (4.7)	2 (16.7)	0.156
MLS; mean ± sd	6.50 ± 6.14	4.20 ± 3.49	10.33 ± 8.50	0.189
Third ventricle compression				
Absent; *n*(%)	42 (76.4)	36 (83.7)	6 (50.0)	0.32
Partial; *n*(%)	10 (18.2)	6 (14.0)	4 (33.3)	
Total; *n*(%)	3 (5.5)	1 (2.3)	2 (16.7)	
Lateral ventricle compression				
Absent; *n*(%)	41 (74.5)	36 (83.7)	5 (41.7)	0.006
Partial; *n*(%)	13 (23.6)	7 (16.3)	6 (50.0)	
Total; *n*(%)	1 (1.8)	0 (0.0)	1 (8.3)	
Cistern compression				
Absent; *n*(%)	44 (80.0)	39 (90.7)	5 (41.7)	0.001
Partial; *n*(%)	7 (12.7)	3 (7.0)	4 (33.3)	
Total; *n*(%)	4 (7.3)	1 (2.3)	3 (25.0)	
Sulcus effacement				
Absent; *n*(%)	38 (69.1)	34 (79.1)	4 (33.3)	0.003
Partial; *n*(%)	13 (23.6)	8 (18.6)	5 (41.7)	
Total; *n*(%)	4 (7.3)	1 (2.3)	3 (25.0)	
Other findings				
EDH; *n*(%)	9 (16.4)	9 (20.9)	0 (0)	0.083
SDH; *n*(%)	18 (32.7)	10 (23.3)	8 (66.7)	0.005
SDH dimension; mean ± sd	6.58 ± 4.17	4.80 ± 2.11	8.81 ± 5.12	0.038
ASH; *n*(%)	26 (47.3)	16 (37.2)	10 (83.3)	0.005
Contusion; *n*(%)	25 (45.5)	18 (41.9)	7 (58.3)	0.311
Depressed skull fracture; *n*(%)	3 (5.5)	2 (4.7)	1 (8.3)	0.619
Pneumocephalus; *n*(%)	8 (14.5)	5 (11.6)	3 (25.0)	0.245

mRS, modified Rankin Scale; IQR, interquartile range; MLS, midline shift; EDH, epidural hematoma; SDH, subdural hematoma; ASH, acute subarachnoid hemorrhage.

**Table 3:** Biomarkers of endothelial glycocalyx (eGC) shedding, endothelial and neuronal injury, vascular permeability, inflammation, and sublingual microcirculation (PBR and MVHS) stratified by the presence (+) or absence (−) of cerebral edema signs on cranial computed tomography (midline shift, third ventricle/lateral ventricle/cistern compression, and sulcal effacement).

Biomarkers	Midline shift			Third ventricle compression			Lateral ventricle compression			Cistern compression			Sulcus effacement		
	−	+	*p*	−	+	*p*	−	+	*p*	−	+	*p*	−	+	*p*
*N*	47	8		42	13		41	14		44	11		38	17	
eGC shedding, median (IQR)															
SDC-1, ng/mL	1,668 (697–11,000)	7,744 (1,684–14,750)	0.201	1,718 (761–12,000)	3,859 (916–13,000)	0.560	1,700 (761–11,000)	5,487 (1,154–16,000)	0.560	1,894 (767–11,500)	1,933 (677–12,000)	0.849	1,718 (761–11,000)	3,859 (916–14,000)	0.465
CD44s, ρg/mL	891 (629–1,219)	968 (830–1,458)	0.214	904 (645–1,219)	969 (727–1,232)	0.751	891 (645–1,219)	959 (727–1,232)	0.742	904 (632–1,221)	948 (727–1,232)	0.752	885 (634–1,167)	969 (733–1,463)	0.353
HA, ρg/mL	12 (9–17)	14 (5–16)	0.615	13 (9–19)	11 (8–15)	0.404	13 (9–19)	11 (6–15)	0.233	13 (9–18)	12 (8–15)	0.569	13 (9–19)	12 (8–15)	0.416
GAGs, μg/mg	0.20 (0.11–0.31)	0.08 (0.05–0.21)	0.188	0.18 (0.07–0.28)	0.23 (0.11–0.31)	0.550	0.19 (0.08–0.30)	0.22 (0.09–0.31)	0.859	0.19 (0.08–0.31)	0.13 (0.08–0.31)	0.659	0.18 (0.08–0.28)	0.21 (0.09–0.31)	0.888
Endothelial cell damage, median (IQR)															
sTM, ρg/mL	701 (562–1,175)	878 (615–1,663)	0.396	687 (580–1,175)	901 (583–1,300)	0.321	689 (580–1,175)	875 (583–1,300)	0.451	692 (571–1,185)	901 (600–1,132)	0.673	687 (562–1,054)	901 (600–1,300)	0.229
Vascular permeability, median (IQR)															
Microalbuminuria, mg/g	17.40 (7.59–69.40)	72.48 (32.42–209.25)	0.063	18.59 (9.40–93.62)	32.42 (7.59–153.97)	0.699	18.27 (9.40–93.62)	36.01 (10.79–113.22)	0.601	17.24 (7.36–52.32)	83.05 (39.60–168.54)	0.017	17.95 (9.40–93.62)	33.49 (7.59–153.97)	0.532
Neuronal injury, median (IQR)															
NSE, ng/mL	2,316 (1,892–3,638)	2,692 (1,993–4,530)	0.550	2,261 (1,892–3,349)	2,802 (2,301–5,323)	0.099	2,254 (1,892–3,318)	3,269 (2,301–5,323)	0.060	2,261 (1,856–3,346)	3,802 (2,301–5,323)	0.104	2,249 (1,721–3,349)	2,802 (2,292–4,491)	0.090
Inflammatory mediator, median (IQR)															
IL-6, ρg/mL	117 (30–300)	177 (70–243)	0.445	117 (30–232)	118 (56–366)	0.362	117 (30–226)	170 (56–366)	0.254	117 (29–226)	118 (56–297)	0.569	117 (30–226)	118 (56–297)	0.402
Sublingual microcirculation, median (IQR)															
Adj PBR, μm	1.56 (1.42–1.74)	1.71 (1.53–2.09)	0.149	1.58 (1.43–1.77)	1.54 (1.42–1.82)	0.799	1.58 (1.42–1.78)	1.54 (1.42–1.82)	0.931	1.56 (1.42–1.79)	1.64 (1.42–1.82)	0.512	1.58 (1.42–1.79)	1.55 (1.43–1.77)	0.902
MVHS points	3.52 (2.72–4.62)	2.94 (2.20–3.60)	0.128	3.07 (2.62–4.16)	3.90 (3.53–4.79)	0.241	3.06 (2.58–4.20)	3.87 (3.31–4.79)	0.247	3.17 (2.62–4.31)	3.53 (2.53–4.62)	0.734	3.05 (2.53–4.16)	3.85 (3.20–4.62)	0.296

IQR, interquartile range; SDC-1, syndecan-1; CD44s, antigen soluble CD44; GAGs, sulfated glycosaminoglycan; HA, hyaluronan; sTM, thrombomodulin; IL-6, interleukin-6; NSE, neuron-specific enolase; Adj PBR, adjusted perfused boundary region; MVHS, microvascular health score.

For neurological prognosis evaluation through the mRS at 3 months after the TBI, only the microalbuminuria levels were significantly higher in patients with poor neurological outcomes [72.48 mg/g (IQR 33.49–156.50)] than in the good neurological outcomes group [17.08 mg/g (IQR 7.17–52.88); *p* = 0.009]. There were no other statistically significant differences between the groups (Table 4 and Figure 4).

**Table 4:** Comparison of biomarkers of endothelial glycocalyx (eGC) shedding, endothelial cell damage, vascular permeability, neuronal injury, inflammatory mediators, and sublingual microcirculation parameters between patients with good and poor neurological outcomes after traumatic brain injury (TBI).

Biomarkers, median (IQR)	Good neurological outcomes (mRS < 4)	Poor neurological outcomes (mRS ≥ 4)	*p*
	*N* = 43	*N* = 12	
eGC shedding			
SDC-1; ng/mL (blood)	2,722(772–12,000)	1,700(408–10,000)	0.357
CD44s; ρg/mL (blood)	891(629–1,223)	959(730–1,228)	0.568
HA; ρg/mL (blood)	12(8–17)	14(10–17)	0.602
GAGs; μg/mg (urine)	0.19(0.07–0.32)	0.20(0.10–0.29)	0.662
Endothelial cell damage			
sTM; ρg/mL (blood)	694(562–1,175)	829(592–1,185)	0.791
Vascular permeability			
Microalbuminuria; mg/g (urine)	17.08(7.17–52.88)	72.48(33.49–156.50)	0.009
Neuronal injury			
NSE; ng/mL (blood)	2,254(1,820–3,349)	3,173(2,280–4,330)	0.136
Inflammatory mediator			
IL-6; ρg/mL (blood)	115(30–300)	144(63–275)	0.463
Sublingual microcirculation			
PVD; mm/mm^2^ × 10^−2^, mean ± sd	481 ± 93	492 ± 91	0.727
RBC filling; (%), median (IQR)	73(68–77)	73(69–75)	0.846
PBR; μm, median (IQR)	1.87(1.73–2.10)	1.82(1.77–2.12)	0.991
Adjusted PBR; μm, median (IQR)	1.56(1.43–1.77)	1.62(1.44–1.80)	0.453
MVHS; points, median (IQR)	3.18(2.62–4.67)	3.58(2.63–4.07)	0.838
Prognostic score			
CRASH; points, median (IQR)	8.30(2.70–18.80)	24.95(9.00–57.40)	0.012

mRS, modified Rankin Scale; IQR, interquartile range; SDC-1, syndecan-1; CD44s, soluble antigen CD44; HA, hyaluronan; GAGs, sulfated glycosaminoglycans; sTM, thrombomodulin; NSE, specific-neuron enolase; IL-6, interleukin-6; PVD, perfused vascular density; RBC, red blood cell; PBR, perfused boundary region; MVHS, microvascular health score; CRASH, Corticosteroid Randomization After Significant Head injury score.

## Discussion

This study provides evidence of early eGC shedding and consequent vascular hyperpermeability after TBI. Elevated microalbuminuria levels were linked to tomographic evidence of cerebral edema (cistern compression) and to poorer neurological outcomes. The baseline characteristics of our cohort were similar to those reported in other TBI studies (27), with the majority of patients being young men (89%), 38 ± 14 years old, most frequently injured in motorcycle accidents (38%), and showing an ISS of 17 ± 9. An ISS above 15 is generally accepted as indicative of severe trauma (17). In our study, 70% of patients had extracranial injuries, consistent with the typical polytrauma profile seen in high-complexity tertiary centers (15). This represents a methodological limitation, as the extent to which the observed eGC shedding can be attributed solely to TBI remains uncertain. Nevertheless, our findings are particularly noteworthy because patients were evaluated in the hyperacute phase of TBI, without circulatory shock or coagulopathy, allowing us to suggest a possible association between eGC shedding and TBI itself. Previous studies have reported isolated elevations of individual eGC shedding biomarkers in TBI, particularly SDC-1 levels. Gonzalez Rodriguez et al. (14, 15) showed an association between elevated SDC-1 levels and mortality in polytrauma patients, while Albert et al. (28) linked increased SDC-1 to coagulopathy in severe trauma. A recent meta-analysis by Xie et al. (29), pooling data from more than one thousand patients, reinforced the association between elevated SDC-1 and mortality; however, these studies were limited to a single biomarker of eGC shedding. Our investigation advances in the field by simultaneously measuring multiple eGC shedding biomarkers and estimating eGC thickness through sublingual microcirculation assessment. We observed a significant increase in microalbuminuria among patients with TBI compared with control subjects. Importantly, the majority of patients were young and lacked comorbidities that might compromise renal function. To the best of our knowledge, only one previous study has investigated microalbuminuria following trauma, reporting elevated levels within the first 24 h post-injury (30). Moreover, we identified a statistically significant correlation between microalbuminuria levels and adjusted PBR, supporting the hypothesis that eGC shedding contributes to the acute vascular hyperpermeability established in this setting. Direct evaluation of BBB permeability was not undertaken due to the methodological challenges associated with *in vivo* assessment (31). Nonetheless, patients exhibiting cistern compression demonstrated higher microalbuminuria levels, suggesting that the systemic vascular hyperpermeability observed in these individuals may also extend to the BBB. Furthermore, we identified a positive correlation between NSE concentrations—used as a surrogate marker of neuronal injury and BBB disruption—and biomarkers of eGC shedding (SDC-1 and CD44s), endothelial injury (sTM), inflammatory activation (IL-6), and microalbuminuria. Collectively, these findings suggest that neuronal injury and BBB dysfunction arise from a multifactorial process involving concurrent endothelial damage, inflammatory responses, and vascular barrier impairment (32, 33). In contrast, parameters of sublingual microcirculation, particularly the adjusted PBR, did not correlate with NSE levels. This lack of association may suggest that sublingual microcirculatory alterations are not representative of the changes taking place in the cerebral microcirculation. Structural CT findings, including sulcal effacement, ventricular compression, and midline shift, were more frequently observed in patients with poorer functional outcomes. Individuals exhibiting CT features consistent with cerebral edema demonstrated higher levels of SDC-1, CD44s, sTM, and microalbuminuria; however, statistical significance was reached only for microalbuminuria in patients with cistern compression. We speculate that if a more sensitive and refined imaging modality—such as brain magnetic resonance imaging (MRI)—had been available, additional associations with these biomarkers might have been detected (18). Unfortunately, MRI was not accessible in our emergency department at the time of data collection. In our cohort, only microalbuminuria levels differentiated patients with unfavorable neurological outcomes at 3 months. However, the primary objective of our study was to elucidate the pathophysiological relationship between eGC shedding and vascular hyperpermeability following TBI. The absence of a statistically significant prognostic association between eGC shedding biomarkers and clinical outcomes may be attributable to the limited sample size and the early timing of biomarker assessment. While this study does not test therapies, it provides a foundation for future studies on preserving or restoring the eGC after TBI. Preclinical interventions studied with this goal include valproic acid, plasma transfusion, hyaluronan hydrogels, and β-blockers, but supportive data in humans are currently insufficient (34–37).

This study has several limitations. It was a single-center study with a small sample size, designed primarily to explore the pathophysiological relationship between eGC shedding and vascular hyperpermeability after TBI. A substantial proportion of participants sustained concomitant extracranial injuries, which may have confounded biomarker interpretation. Furthermore, biomarkers were assessed at a single early time point, precluding evaluation of temporal trends. Sublingual microcirculation may not accurately represent cerebral microvascular physiology, limiting the interpretation of PBR and MVHS data. Similarly, microalbuminuria reflects renal vascular permeability and may not directly correspond to BBB integrity. Finally, the direct assessment of BBB permeability was not performed due to methodological complexity and cost constraints.

In conclusion, the integration of multiple circulating biomarkers with sublingual microcirculation imaging provides evidence that eGC shedding takes place during the early phase of TBI, promoting vascular hyperpermeability and the onset of vasogenic cerebral edema.

## Data Availability

The raw data supporting the conclusions of this article will be made available by the authors, without undue reservation.

## References

[1] Dewan MC Rattani A Gupta S Baticulon RE Hung YC Punchak M . Estimating the global incidence of traumatic brain injury. J Neurosurg. (2019) 130:1080–97. doi: 10.3171/2017.10.JNS17352, 29701556

[2] Dobson GP Morris JL Letson HL. Traumatic brain injury: symptoms to systems in the 21st century. Brain Res. (2024) 1845:149271. doi: 10.1016/j.brainres.2024.149271, 39395646

[3] Werner C Engelhard K. Pathophysiology of traumatic brain injury. Br J Anaesth. (2007) 99:4–9. doi: 10.1093/bja/aem131, 17573392

[4] Cardona-Collazos S Gonzalez WD Pabon-Tsukamoto P Gao GY Younsi A Paiva WS . Cerebral Edema in traumatic brain injury. Biomedicine. (2025) 13:1–18. doi: 10.3390/biomedicines13071728, 40722798 PMC12292430

[5] Maas AIR Menon DK Adelson PD Andelic N Bell MJ Belli A . Traumatic brain injury: integrated approaches to improve prevention, clinical care, and research. Lancet Neurol. (2017) 16:987–1048. doi: 10.1016/S1474-4422(17)30371-X, 29122524

[6] Reitsma S Slaaf DW Vink H van Zandvoort MA oude Egbrink MG. The endothelial glycocalyx: composition, functions, and visualization. Pflugers Arch Eur J Physiol. (2007) 454:345–59. doi: 10.1007/s00424-007-0212-8, 17256154 PMC1915585

[7] Gomez Toledo A Golden GJ Cummings RD Malmstrom J Esko JD. Endothelial glycocalyx turnover in vascular health and disease: rethinking endothelial dysfunction. Annu Rev Biochem. (2025) 94:561–86. doi: 10.1146/annurev-biochem-032620-104745, 40132227 PMC13377631

[8] Ando Y Okada H Takemura G Suzuki K Takada C Tomita H . Brain-specific ultrastructure of capillary endothelial glycocalyx and its possible contribution for blood brain barrier. Sci Rep. (2018) 8:17523. doi: 10.1038/s41598-018-35976-2, 30504908 PMC6269538

[9] Zhao F Zhong L Luo Y. Endothelial glycocalyx as an important factor in composition of blood-brain barrier. CNS Neurosci Ther. (2021) 27:26–35. doi: 10.1111/cns.13560, 33377610 PMC7804892

[10] Chelazzi C Villa G Mancinelli P De Gaudio AR Adembri C. Glycocalyx and sepsis-induced alterations in vascular permeability. Crit Care. (2015) 19:26. doi: 10.1186/s13054-015-0741-z, 25887223 PMC4308932

[11] Chignalia AZ Yetimakman F Christiaans SC Unal S Bayrakci B Wagener BM . The glycocalyx and trauma: a review. Shock. (2016) 45:338–48. doi: 10.1097/SHK.0000000000000513, 26513707 PMC4792653

[12] Chen Y Gu M Patterson J Zhang R Statz JK Reed E . Temporal alterations in cerebrovascular glycocalyx and cerebral blood flow after exposure to a high-intensity blast in rats. Int J Mol Sci. (2024) 25:1–16. doi: 10.3390/ijms25073580, 38612392 PMC11011510

[13] Hall AA Mendoza MI Zhou H Shaughness M Maudlin-Jeronimo E McCarron RM . Repeated low intensity blast exposure is associated with damaged endothelial glycocalyx and downstream behavioral deficits. Front Behav Neurosci. (2017) 11:104. doi: 10.3389/fnbeh.2017.00104 28649193 PMC5465256

[14] Gonzalez Rodriguez E Ostrowski SR Cardenas JC Baer LA Tomasek JS Henriksen HH . Syndecan-1: a quantitative marker for the endotheliopathy of trauma. J Am Coll Surg. (2017) 225:419–27. doi: 10.1016/j.jamcollsurg.2017.05.012 28579548

[15] Gonzalez Rodriguez E Cardenas JC Cox CS Kitagawa RS Stensballe J Holcomb JB . Traumatic brain injury is associated with increased syndecan-1 shedding in severely injured patients. Scand J Trauma Resusc Emerg Med. (2018) 26:102. doi: 10.1186/s13049-018-0565-3, 30463625 PMC6249764

[16] Collaborators MCT Perel P Arango M Clayton T Edwards P Komolafe E Poccock S . Predicting outcome after traumatic brain injury: practical prognostic models based on large cohort of international patients. BMJ. (2008) 336:425–9. doi: 10.1136/bmj.39461.643438.25 18270239 PMC2249681

[17] Baker SP O'Neill B Haddon W Jr Long WB. The injury severity score: a method for describing patients with multiple injuries and evaluating emergency care. J Trauma. (1974) 14:187–96. doi: 10.1097/00005373-197403000-00001, 4814394

[18] Kim JJ Gean AD. Imaging for the diagnosis and management of traumatic brain injury. Neurotherapeutics. (2011) 8:39–53. doi: 10.1007/s13311-010-0003-3, 21274684 PMC3026928

[19] Marshall LF Marshall SB Klauber MR Van Berkum Clark M Eisenberg H Jane JA . The diagnosis of head injury requires a classification based on computed axial tomography. J Neurotrauma. (1992) 9:S287–92. 1588618

[20] Cheng F Yuan Q Yang J Wang W Liu H. The prognostic value of serum neuron-specific enolase in traumatic brain injury: systematic review and meta-analysis. PLoS One. (2014) 9:e106680. doi: 10.1371/journal.pone.0106680, 25188406 PMC4154726

[21] Lu W Jiang C Wang Z Chen Y Bai R Yan G . Lactic acid, neuron-specific enolase, and blood-brain barrier index after a severe traumatic brain injury: a prospective study. Br J Neurosurg. (2024) 38:220–4. doi: 10.1080/02688697.2020.1823938, 33016150

[22] Schmidt EP Overdier KH Sun X Lin L Liu X Yang Y . Urinary glycosaminoglycans predict outcomes in septic shock and acute respiratory distress syndrome. Am J Respir Crit Care Med. (2016) 194:439–49. doi: 10.1164/rccm.201511-2281OC, 26926297 PMC5003330

[23] Spapen HD Diltoer MW Nguyen DN Hendrickx I Huyghens LP. Effects of N-acetylcysteine on microalbuminuria and organ failure in acute severe sepsis: results of a pilot study. Chest. (2005) 127:1413–9. doi: 10.1378/chest.127.4.1413, 15821223

[24] Nieuwdorp M Mooij HL Kroon J Atasever B Spaan JA Ince C . Endothelial glycocalyx damage coincides with microalbuminuria in type 1 diabetes. Diabetes. (2006) 55:1127–32. doi: 10.2337/diabetes.55.04.06.db05-1619, 16567538

[25] Rovas A Sackarnd J Rossaint J Kampmeier S Pavenstadt H Vink H . Identification of novel sublingual parameters to analyze and diagnose microvascular dysfunction in sepsis: the NOSTRADAMUS study. Crit Care. (2021) 25:112. doi: 10.1186/s13054-021-03520-w, 33741036 PMC7980588

[26] Janssen PM Visser NA Dorhout Mees SM Klijn CJ Algra A Rinkel GJ. Comparison of telephone and face-to-face assessment of the modified Rankin scale. Cerebrovasc Dis. (2010) 29:137–9. doi: 10.1159/000262309, 19955737

[27] CRASH-3 Trial Collaborators. Effects of tranexamic acid on death, disability, vascular occlusive events and other morbidities in patients with acute traumatic brain injury (CRASH-3): a randomised, placebo-controlled trial. Lancet. (2019) 394:1713–23. doi: 10.1016/S0140-6736(19)32233-0, 31623894 PMC6853170

[28] Albert V Subramanian A Agrawal D Pati HP Gupta SD Mukhopadhyay AK. Acute traumatic endotheliopathy in isolated severe brain injury and its impact on clinical outcome. Med Sci (Basel). (2018) 6:1–14. doi: 10.3390/medsci6010005, 29337920 PMC5872162

[29] Xie WW Ding YJ Bhandari S Li H Chen HS Jin SW . Clinical value of Syndecan-1 levels in trauma brain injury: a meta-analysis. Shock. (2024) 61:49–54. doi: 10.1097/SHK.0000000000002255, 37878479

[30] De Gaudio AR Spina R Di Filippo A Feri M. Glomerular permeability and trauma: a correlation between microalbuminuria and injury severity score. Crit Care Med. (1999) 27:2105–8. doi: 10.1097/00003246-199910000-00004 10548189

[31] Moyaert P Padrela BE Morgan CA Petr J Versijpt J Barkhof F . Imaging blood-brain barrier dysfunction: a state-of-the-art review from a clinical perspective. Front Aging Neurosci. (2023) 15:1132077. doi: 10.3389/fnagi.2023.1132077, 37139088 PMC10150073

[32] Caceres E Divani AA Olivella-Gomez J Di Napoli M Reyes LF. Interleukin-6 and its association with outcome in traumatic brain injury: a prospective cohort. Scand J Trauma Resusc Emerg Med. (2025) 33:131. doi: 10.1186/s13049-025-01430-2, 40713822 PMC12296603

[33] Serban NL Ungureanu G Florian IS Ionescu D. Cerebral vascular disturbances following traumatic brain injury: pathophysiology, diagnosis, and therapeutic perspectives-a narrative review. Life. (2025) 15:1–23. doi: 10.3390/life15091470, 41010412 PMC12471948

[34] Jepsen CH deMoya MA Perner A Sillesen M Ostrowski SR Alam HB . Effect of valproic acid and injury on lesion size and endothelial glycocalyx shedding in a rodent model of isolated traumatic brain injury. J Trauma Acute Care Surg. (2014) 77:292–7. doi: 10.1097/TA.0000000000000333 25058256

[35] Kravitz MS Kattouf N Stewart IJ Ginde AA Schmidt EP Shapiro NI. Plasma for prevention and treatment of glycocalyx degradation in trauma and sepsis. Crit Care. (2024) 28:254. doi: 10.1186/s13054-024-05026-7, 39033135 PMC11265047

[36] Liu X Wu C Zhang Y Chen S Ding J Chen Z . Hyaluronan-based hydrogel integrating exosomes for traumatic brain injury repair by promoting angiogenesis and neurogenesis. Carbohydr Polym. (2023) 306:120578. doi: 10.1016/j.carbpol.2023.120578, 36746568

[37] Hart S Lannon M Chen A Martyniuk A Sharma S Engels PT. Beta blockers in traumatic brain injury: a systematic review and meta-analysis. Trauma Surg Acute Care Open. (2023) 8:e001051. doi: 10.1136/tsaco-2022-001051, 36895782 PMC9990673

